# Tracheal or bronchial wedge resection: Case report

**DOI:** 10.3389/fsurg.2023.1122075

**Published:** 2023-02-14

**Authors:** Zhenhua Jiao, Zhe Tang, Jun Yu

**Affiliations:** Department of Thoracic Surgery, Tongji Hospital, Huazhong University of Science and Technology, Wuhan, China

**Keywords:** tracheal or bronchial tumor, tracheal or bronchial wedge resection, videoassisted thoracoscopic surgery, parenchymal sparing procedure, case report

## Abstract

**Background:**

Primary tracheal or bronchial tumors are relatively uncommon, whether benign or malignant. Sleeve resection is an excellent surgical technique for most primary tracheal or bronchial tumors. However, depending on the size and location of the tumor, thoracoscopic wedge resection of trachea or bronchus can be performed with the assistance of a fiberoptic bronchoscope for some malignant and benign tumors.

**Case Description:**

We performed a single incision video-assisted bronchial wedge resection in a patient with a left main bronchial hamartoma with a size of 7 × 5 × 5 mm. The patient was discharged from the hospital six days after the surgery with no postoperative complications. There was no obvious discomfort during the 6-month postoperative follow-up, and the reexamination of fiberoptic bronchoscopy revealed no evident stenosis of the incision.

**Conclusions:**

Through the detailed case study and literature review, we believe that tracheal or bronchial wedge resection is a significantly superior technique under the appropriate conditions. Video-assisted thoracoscopic wedge resection of trachea or bronchus should be a new and excellent development direction of minimally invasive bronchial surgery.

## Introduction

Primary tracheal or bronchial tumors are relatively uncommon, whether benign or malignant. Tracheal or bronchial tumors are classified into three types: malignant, low-grade, and benign on their degree of differentiation. Tracheal or bronchial segmental resection with end-to-end anastomosis is currently the standard surgical treatment for tracheal or bronchial tumors. Sleeve resection is an excellent surgical technique for most primary tracheal or bronchial tumors. However, depending on the size and location of the tumor, thoracoscopic wedge resection of trachea or bronchus can be performed with the assistance of a fiberoptic bronchoscope for some malignant and benign tumors (1, 2). It significantly reduces the difficulty and trauma of surgery while preserving the lung tissue and ensuring surgical results. It also reduces the incidence of postoperative complications (3–8). This article comprehensively demonstrates the advantages, disadvantages and indications of this technique through a detailed case study and previous literature.

## Case description

**Figure 1:**
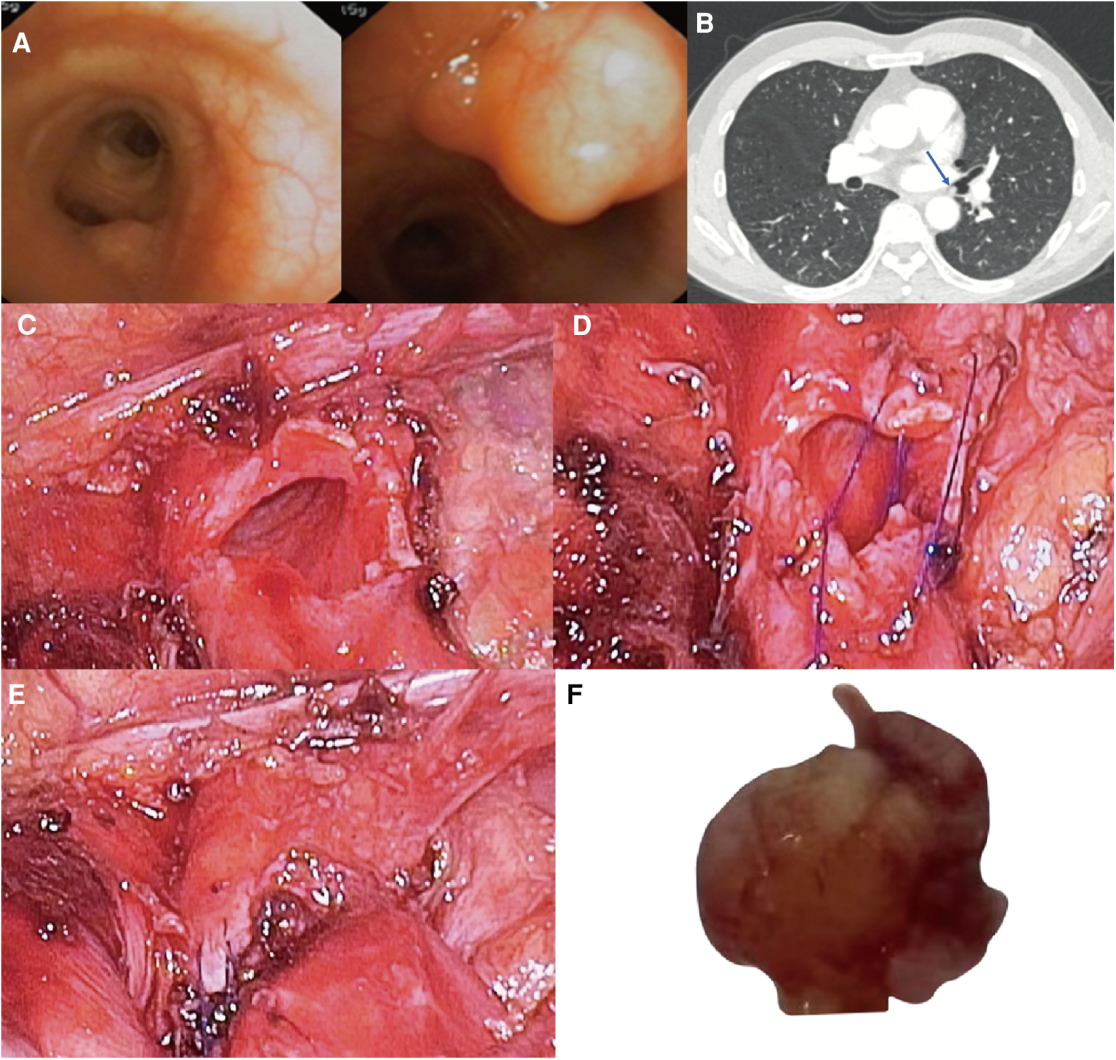
(**A**) neoplastic bulge was seen on the medial wall of left main bronchus terminal, 1 cm away from the opening of left lower lobe bronchus. (**B**) Bronchial tumor at the end of left main bronchus (arrow), the diameter of tumor base is 5 mm, and the diameter of bronchus is 8 mm. Surgical Technique: (**C**) The left main bronchus wall after wedge-shaped resection of the tumor with endoscopic scissors. (**D**) Continuous suture of the incision using 4–0 Prolene. (**E**) Left main bronchus wall after suture. (**F**) Left main bronchus tumor with a size of 7 × 5 × 5 mm.

A 46-year-old man was admitted with a left main bronchus tumor. He was in good health in the past. Physical examination, routine blood examination, biochemistry and tumor markers revealed no abnormalities. After admission, fiberoptic bronchoscopy (Figure 1) and chest computed tomography (CT) (Figure 1) were performed. At the bronchoscopy, a certain amount of tissue was taken for biopsy, although we found that the tumor was tough and difficult to clamp.

Bronchoscopy results revealed a neoplastic bulge on the medial wall of left main bronchus terminal, 1 cm away from the left lower lobe bronchus opening. The surface mucosa was still smooth, and the surface blood vessels were visible. The histopathological results of the biopsy showed that there were very small pieces of proliferated spindle cells with background myxoid changes under microscope, and the cell heterogeneity was not obvious. Although there was no definite diagnosis, it also helped us to preliminarily rule out the diagnosis of malignant tumor. In this case, we first communicated with a respiratory endoscopist and were told that the tumor could not be safely removed by intraluminal bronchoscopic treatment due to the large basal area of the tumor, so we decided to perform a single incision video-assisted bronchial wedge resection first.

## Surgical technique

We drilled a 3 cm left thoracic and axillary midline fifth intercostal hole into the patient’s chest. After loosening some adhesions and dividing the inferior pulmonary ligament, the lung tissue was pulled to expose the left main bronchus from the back. The left main bronchus was separated after the mediastinal pleura was opened. At this time, the visual fiberoptic bronchoscope was used to determine tumor location and margin by two methods: (1) We asked the anesthesiologist to place the bronchoscope lens under the tumor and turn the lens direction so that the light source of the lens was directed directly at the bronchial wall. And then we could clearly see the position of the lens under the thoracoscopy. (2) We pressed the bronchus gently with the instrument under the thoracoscopy, and the part we pressed could be clearly seen under the fiberoptic bronchoscope. By comparing these two noninvasive methods, we could determine the location of the tumor.

**Figure 2:**
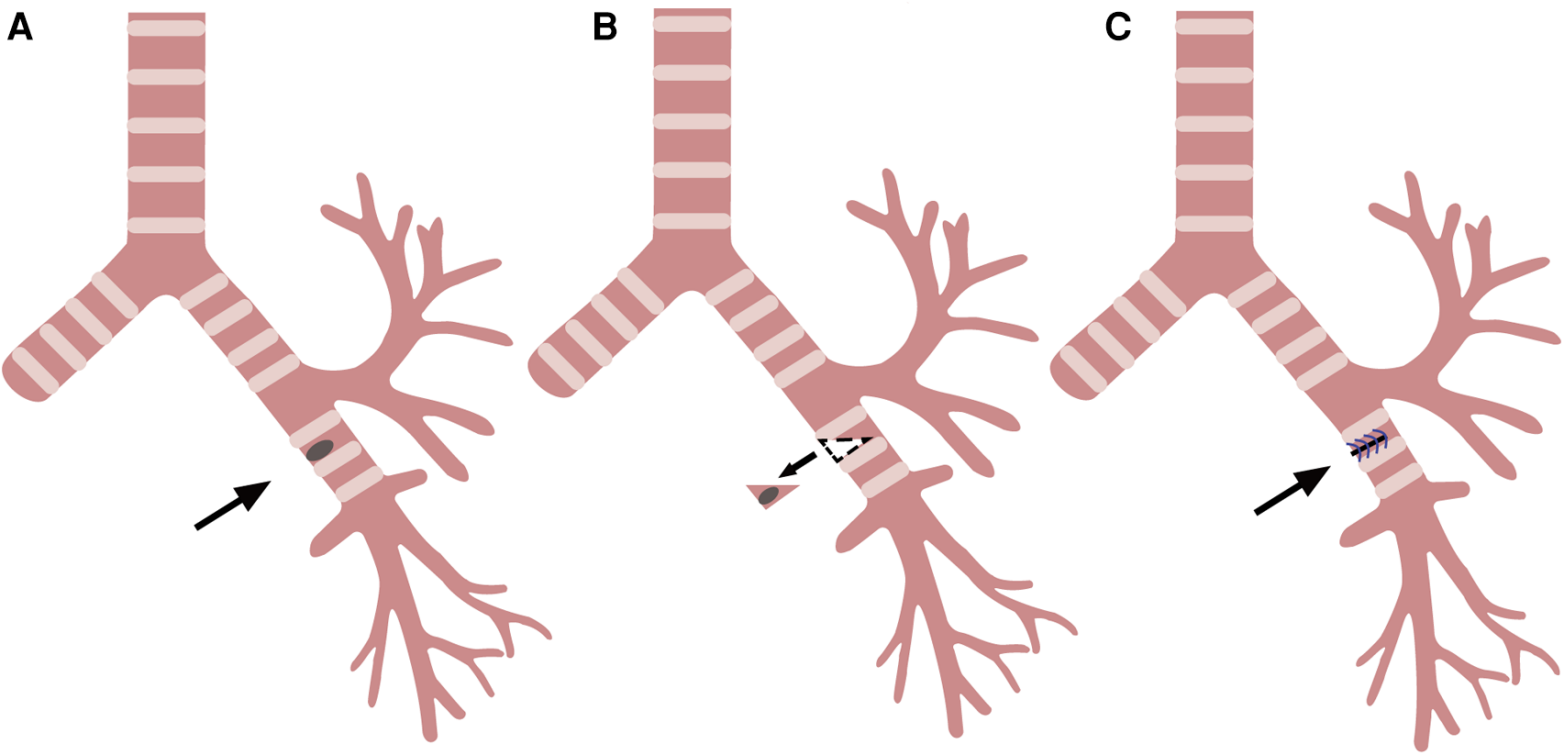
Wedge resection and reconstruction of the bronchi. (**A**) Left main bronchus tumor (arrow). (**B**) Resection of the tumor along the dotted line (arrow). (**C**) Left main bronchus wall after suture (arrow).

After the bronchial tumor was leaked, the left main bronchus was cut along the distal end of tumor with endoscopic scissors, and the tumor was wedge-shaped along the edge of tumor. When the left main bronchial hamartoma was confirmed from the frozen section of the removed tumor, the incision was sutured by 4 - 0 Prolene with a continuous suture, needle distance 5 mm, and margin 5 mm (Figures 1, 2). There was no apparent stenosis or distortion of the bronchi observed by thoracoscopy and fiberoptic bronchoscopy after suturing. The operation lasted 1.5 h, and there was no visible bleeding during surgery.

The chest drain was removed five days after the surgery, and the patient was discharged from the hospital six days after the surgery with no postoperative complications. Thoracoscopic wedge resection is significantly superior to sleeve resection in terms of recovery. There was no obvious discomfort during the 6-month postoperative follow-up, and the reexamination of fiberoptic bronchoscopy revealed no evident stenosis of the incision.

## Discussion

Through the detailed case study and literature review, we believe that tracheal or bronchial wedge resection is feasible and excellent in treating some benign and malignant tumors of the trachea or bronchus. The indications for wedge resection include (1) Tumors with a base diameter smaller than the diameter of trachea or bronchus. (2) Tumors that are confined to the carina or bronchial corner. (3) Local tumor infiltration in the cranial and the caudal parts of adjoining main bronchus.

### Indication 1

In the 6 cases in the Supplementary Table (9–13), the authors performed wedge resection of the trachea or bronchus to treat benign or low-grade bronchial tumors. Wedge resection significantly reduces the difficulty and risk of surgery while preserving the lung tissue and ensuring surgical results. Based on their experience with 83 cases of bronchial carcinoid tumors, Ismail Cüneyt Kurul et al. concluded that wedge resection could be considered if the diameter of the trachea or bronchial tumor base to be resected is smaller than the diameter of bronchus (2). In addition, Florian Augustin believe that the maximum distance between the upper and lower edge of the bronchus in wedge resection is preferably no longer than the transverse diameter of the bronchus (14), which can effectively avoid postoperative anastomotic stenosis. For benign and low-grade malignant tumors of the trachea or bronchus, the preferred surgical approach should be minimally invasive thoracoscopic wedge resection.

### Indication 2

**Figure 3:**
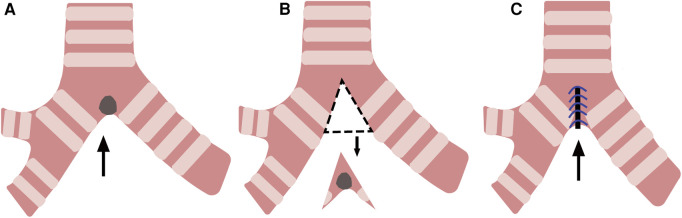
Wedge resection and reconstruction of the carina. (**A**) A tumor at the carina(arrow). (**B**) Resection of the tumor along the dotted line (arrow). (**C**) Reconstruction of the carina (arrow).

Dong Xie et al. performed wedge resection on a patient with a 1 cm squamous cell carcinoma confined to the tracheal carina, and a clear margin was confirmed during the surgery. The wedge-shaped excision and reconstruction of the carina under the original carina ensured no separation between the trachea and the main bronchus (Figure 3). The trachea and the bronchus remained continuous without creating longitudinal tension. The problem of longitudinal tension encountered by sleeve resection was skillfully circumvented with wedge resection, and the patient recovered well after surgery (15). Hiromasa Yamamoto et al. performed a bronchial wedge resection for a carcinoid tumor of the left upper bronchus near the upper and lower lobar bronchi bifurcation. The upper and healthy lower lobar bronchial corner was resected longitudinally, and the bronchial corner was reconstructed at a distance. At the five-month postoperative follow-up, there was no stenosis at the suture, indicating that this technique avoided anastomotic stenosis and longitudinal tension (16).

Daisuke Yuki et al. performed a deeper wedge resection and the reconstruction of right secondary carina for a 13 mm recurrent mucoepidermoid carcinoma located at the orifice of upper lobar bronchus with main bronchus involvement. The lung tissue was successfully preserved, and no recurrence occurred 18 months after surgery (17). Dong Xie, Hiromasa Yamamoto, and Daisuke Yuki performed wedge resection and reconstruction of the carina, bronchial corner, and the secondary carina, respectively, for resection of tracheal or bronchial malignancies. The technique avoided longitudinal tension and anastomosis stenosis and preserved the lung tissue intact.

### Indication 3

Krishna Khargi et al. performed lobectomy with bronchial wedge resection in eight patients with lung malignancies involving the main bronchus, including four right upper lobectomies, two left upper lobectomies, and two left lower lobectomies. Postoperative histopathological results revealed seven cases of squamous cell carcinoma and one case of carcinoid. They believe it is feasible to remove one-third to one-half of the circumference of the main bronchus in the wedge resection (1). However, three patients experienced varying degrees of bronchial stenosis after the operation. Therefore, Florian Augustin et al. suggested that the maximum distance between the upper and lower edges of the bronchus in wedge resection should be less than the transverse diameter of the bronchus, which is more conducive to avoiding anastomotic stenosis (14).

In 16 patients with right lung malignancies, Christophoros Kotoulas et al. performed 12 right upper lobectomies and four right upper and middle lobectomies combined with main bronchial wedge resection. They dissected the inferior pulmonary ligament and released the hilum, allowing the trachea and main bronchi to move 1–2 cm (6). None of the 16 patients had anastomotic stenosis and distortion after surgery, and the long-term prognosis was satisfactory.

**Figure 4:**
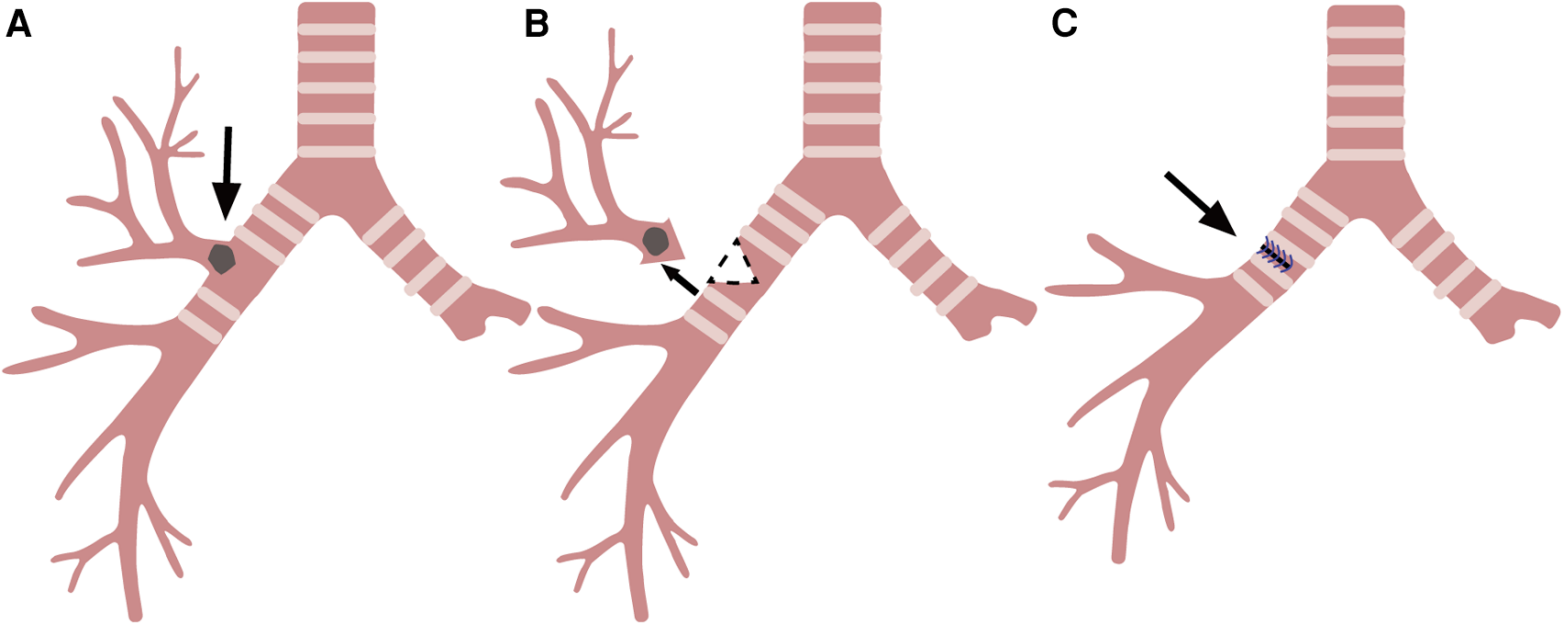
Right upper lobectomy with wedge resection and reconstruction of the bronchus. (**A**) Right upper lung malignancy involving the main bronchus (arrow). (**B**) Resection of the right superior lobar bronchus along the dotted line (arrow). (**C**) Right main bronchus wall after suture (arrow).

Although Krishna Khargi et al. thought that local tumor infiltration of the cranial and the caudal parts of adjoining main bronchus was the indication for wedge bronchoplasty (1), the use of wedge resection has many limitations. First, although the wedge bronchoplasty can be performed on either lobe, the right upper lobe is more suitable for anatomical reasons (6, 18) (Figure 4). In addition, the mobilization of the trachea and main bronchus, the limitation of resection range of main bronchus, and the determination of resection margin are all necessary to ensure the safety of wedge bronchoplasty.

Similarly, for benign tracheal or bronchial tumors, we recommend lobectomy or segmentectomy with bronchial wedge resection rather than sleeve resection if the obstruction of the bronchus has resulted in irreversible destruction of the lung tissue and lobectomy or segmentectomy alone cannot resolve the problem. For example, Azevedo-Pereira AE (19), Galvez C (20), and Maeda M (21) used lobectomy or segmentectomy with wedge bronchoplasty for the treatment of bronchial glomus tumor, bronchial lipomas, and bronchial inflammatory pseudotumors, respectively.

These demonstrate that tracheal or bronchial wedge resection is a feasible and excellent technique when the indications for wedge resection are understood, particularly for some benign and malignant tumors with guaranteed margins. The indications for wedge resection include (1) Tumors with a base diameter smaller than the diameter of trachea or bronchus. (2) Tumors that are confined to the carina or bronchial corner. (3) Local tumor infiltration in the cranial and the caudal parts of adjoining main bronchus.

In addition, wedge resection requires the dissection of the inferior pulmonary ligament, the hilum release, the dissociation of intrapericardial pulmonary vein attachments, the mobilization of the trachea and main bronchus, the limitation of resection range, the determination of resection margin, and suturing from low tension area to high tension area. These are effective measures for preventing anastomotic stenosis and ensuring the safety of wedge resection.

Compared with sleeve resection, wedge resection preserves the continuity and blood supply of the trachea or bronchus (22, 23). It significantly reduces the difficulty and trauma of surgery and is easier to be performed under thoracoscopy without conversion to thoracotomy (22). It also reduces the incidence of postoperative complications such as bronchopleural fistula (24). For benign and low-grade malignant tumors of the trachea or bronchus, the preferred surgical approach should be video-assisted thoracoscopic wedge resection. For tumors such as non-small cell lung cancer, Park et al. found that wedge bronchoplastic lobectomy should be an appropriate alternative to sleeve lobectomy regardless of lymph node status (23).

However, we found that there is a debate about which technique is more likely to cause postoperative anastomotic complications. Although Krüger et al. believe that sleeve resection is more prone to result in anastomotic complications and pneumonia (24), many believe that wedge resection is more prone to result in various degrees of anastomotic stenosis (1, 2). Anastomotic stenosis can cause postoperative complications such as secretion retention, pneumonia, atelectasis, respiratory distress, and complete anastomotic obstruction (1–8, 14, 22, 23). It may result in the patient requiring bronchoscopic toileting or mechanical ventilation support after surgery (1, 23). When stricture is severe, a second operation is required to perform sleeve resection to relieve the anastomotic stenosis (6). However, according to our references, anastomotic stenosis after wedge resection is more of a technical problem. When the indications and precautions of wedge resection are strictly grasped, the probability of anastomotic stenosis after wedge resection is no greater than after sleeve resection.

In conclusion, we believe that tracheal or bronchial wedge resection is a significantly superior technique under the appropriate conditions. Video-assisted thoracoscopic wedge resection of trachea or bronchus should be a new and excellent development direction of minimally invasive bronchial surgery.

## Data Availability

The original contributions presented in the study are included in the article/Supplementary Material, further inquiries can be directed to the corresponding author/s.
